# Palmar Fascia Fibrosis in Dupuytren’s Disease: A Narrative Review of Pathogenic Mechanisms and Molecular Insights

**DOI:** 10.3390/ijms27010382

**Published:** 2025-12-30

**Authors:** Carmelo Pirri

**Affiliations:** Department of Neurosciences, Institute of Human Anatomy, University of Padua, 35121 Padova, Italy; carmelo.pirri@unipd.it

**Keywords:** Dupuytren’s disease, fibrosis, palmar fascia, myofibroblast, transforming growth factor β, extracellular matrix, epigenetics

## Abstract

Dupuytren’s disease (DD) is a chronic fibroproliferative disorder of the palmar fascia, leading to disabling digital contractures and high recurrence after surgery. This narrative review highlights DD as a multifactorial condition in which genetic predisposition and cytogenetic instability converge with extracellular matrix remodeling, aberrant transforming growth factor β (TGF-β) and Wnt/β-catenin signaling, cytoskeletal stabilization and immune-inflammatory amplification. Epigenetic dysregulation further locks fibroblasts into a persistent myofibroblast state. Discrepancies between studies are largely explained by disease stage and experimental anti-inflammatory, antifibrotic and epigenetic strategies to achieve durable disease modification.

## 1. Introduction

Dupuytren’s disease (DD) is a prevalent, progressive fibroproliferative disorder of the palmar fascia, affecting approximately 5–30% of the Northern European population, with significant functional and occupational impact in advanced stages [1]. It is clinically characterized by the development of nodules and collagen-rich cords in the palmar aponeurosis, resulting in progressive flexion contractures of the digits and limited hand function [1]. While the pathognomonic role of myofibroblasts and type I/III collagen accumulation has been extensively described, growing incidence highlights a more complex pathogenic architecture involving immunological activation, progenitor cell reprogramming, mitochondrial dysfunction and redox imbalance [2,3].

Histopathological studies of DD tissue reveal an active fibrotic environment, with elevated expression of α-smooth muscle actin (α-SMA), transforming growth factor-β1 (TGF-β1), fibronectin and tenascin C, paralleling features seen in systemic fibrotic diseases such as scleroderma and idiopathic pulmonary fibrosis [4].

Molecular analysis has uncovered aberrant activation of TGF-β/Smad, Wnt/β-catenin and platelet-derived growth factor (PDGF) signaling pathways, alongside altered expression of profibrotic mediators such as Plasminogen Activator Inhibitor-1 (PAI-1), Insulin-like Growth Factor 2 (IGF2) and Wilms Tumor 1 (WT1) [5,6,7].

Of particular importance is that DD is increasingly viewed not as an isolated fascial disorder but as a focal failure of connective tissue homeostasis, driven by both local mechanical forces and systemic predisposing factors. Genetic studies have identified over 26 susceptibility loci associated with DD, including those linked to WNT signaling (e.g., Wnt Family Member 4 = WNT4, Secreted Frizzled-Related Protein 4 = SFRP4 and R-spondin 2 = RSPO2) and extracellular matrix (ECM) remodeling [8,9].

At the tissue level, palmar fascia exhibits signs of immune cell infiltration, including CD45+, CD68+ macrophages and Langerhans cells, as well as evidence of mitochondrial oxidative stress and dysregulated fibroblast metabolism [10,11].

Recent single-cell transcriptomic data have refined our understanding of stromal heterogeneity in DD, identifying PDPN+, FAP+ and TNFRSF12A+ mesenchymal subsets with high fibrogenic potential [12]. These populations are absent in non-pathological palmar fascia, suggesting that DD arises from a unique confluence of immune, genetic and tissue-intrinsic factors. Additionally, studies of macroscopically normal fascia adjacent to DD lesions reveal upregulation of CTGF, syndecan-1 and FGFR2, indicating the presence of a broader prefibrotic field effect [13].

From a regenerative medicine standpoint, the palmar fascia offers a rare opportunity to investigate fibrosis in a mechanically loaded, surgically accessible connective tissue. Insights from DD research are increasingly relevant to other localized fibrotic conditions, such as Peyronie’s disease, plantar fibromatosis and adhesive capsulitis, underscoring the need to consolidate current knowledge into a unified pathophysiological framework.

The aim of this narrative review is to critically examine the existing literature on DD, focusing on fibrosis and the palmar fascia as the central anatomical and biological substrate. By systematically analyzing experimental, histological, molecular and clinical data, we aim to identify the core mechanisms driving fibrogenesis in DD, highlight converging pathogenic pathways and evaluate emerging therapeutic implications. This synthesis may serve to define not only novel molecular targets for intervention but also to reframe DD as a model of site-specific, immune-regulated fibrosis within the broader spectrum of fascial pathologies.

## 2. Materials and Methods

This narrative review aimed to integrate and critically discuss current evidence on the molecular, histopathological and clinical features of palmar fascia fibrosis in DD. A comprehensive literature search was conducted in PubMed, Scopus and Web of Science to identify peer-reviewed studies published up to August 2025. The search strategy combined the terms “Dupuytren” AND “fibrosis” AND “palmar fascia” and was complemented by related terms (e.g., palmar aponeurosis and palmar aponeurotic fibrosis), manual searches of reference lists and targeted screening of relevant journals. Studies were included if they were written in English and addressed molecular, histopathological or clinical aspects of palmar fascia fibrosis specific to DD, regardless of study design (randomized, observational, preclinical or case reports/series). Exclusion criteria were non-original papers (editorials and commentaries), studies unrelated to the palmar fascia and those focused on post-traumatic or systemic fibrotic disorders not specific to DD. Given the narrative nature of this paper, no formal risk of bias assessment was performed; instead, studies were interpreted qualitatively based on their design, clarity of methods and internal consistency of findings. A narrative synthesis was then carried out to identify recurring concepts, mechanistic pathways and translational implications across biological and clinical domains.

## 3. Genetic and Cytogenetic Contributions

A considerable body of evidence highlights the role of chromosomal abnormalities and genetic instability in DD. Cytogenetic studies, beginning with the work of Bonnici et al. [14], demonstrated that trisomy 8 was present in more than half of the examined DD fibroblast cultures, with additional cases also exhibiting the anomaly in carpal tunnel syndrome controls. This finding provided early support for the hypothesis that genomic instability acts as a predisposing factor in the fibroproliferative process of the palmar fascia. Subsequent investigations expanded these observations, identifying trisomy 7, deletions of chromosomes 6 and 11 and structural rearrangements in fibroblasts derived from Dupuytren’s nodules [8,15,16,17]. While not entirely consistent across studies, these abnormalities suggested that clonal expansion of genetically altered fibroblasts may underlie the nodular proliferation characteristic of DD. Beyond structural chromosomal changes, several authors investigated genetic predisposition and heritability. Familial clustering of DD cases, in particular in Northern European populations, has been well-documented [18]. Linkage studies implicated regions on chromosomes 16q and 6p, although no single causative gene has been definitively established [19]. More recent genome-wide association studies have identified susceptibility loci in Wnt signaling and extracellular matrix-related genes, underscoring the convergence of genetic risk with molecular pathways subsequently confirmed in functional studies [20]. Recent genome-wide association studies (GWASs) have expanded this perspective by identifying more than 26 susceptibility loci associated with DD, several of which cluster in pathways regulating Wnt signaling and ECM homeostasis [21]. Notably, variants near WNT4, SFRP4 and RSP2 have been robustly associated with disease susceptibility, linking germline predisposition directly to canonical fibrogenic pathways [8,9]. These findings reinforce the notion that genetic risk factors in DD converge with the molecular signaling abnormalities observed in fibroblasts, thereby uniting inherited susceptibility with disease pathophysiology. Taken together, these findings suggest that DD is not simply a localized fibrotic lesion but a genetically influenced disorder in which somatic chromosomal instability and inherited susceptibility converge. Cytogenetic instability may drive fibroblast proliferation and ECM deposition, while germline predisposition shapes the population at risk. The overlap between chromosomal abnormalities observed in DD and those seen in other fibroproliferative disorders (e.g., keloids and Peyronie’s disease) further suggest a shared architecture of pathological fibrosis [15] (Table 1).

## 4. Extracellular Matrix (ECM) Remodeling

ECM plays a pivotal role in the pathogenesis of DD, acting not only as a structural scaffold but also as a dynamic regulator of fibroblast activity. Numerous studies included in this review demonstrated that the biochemical composition and biomechanical properties of the ECM directly influence the fibroblast phenotype, thereby contributing to the persistence of fibrosis. In vitro experiments have shown that fibroblast derived from DD cords exhibit enhanced collagen gel contraction compared to control palmar fascia cells, reflecting their heightened contractile potential [22,23]. This increased contractility is tightly linked to the accumulation of type I and III collagen in diseased tissue, which alters both stiffness and architecture of the palmar fascia. The remodeled ECM, in turn, reinforces fibroblast activation, creating a self-perpetuating cycle of fibrosis [24,25]. A particularly notable finding was that type I collagen suppresses β-catenin accumulation while modulating α-smooth muscle actin (α-SMA) expression in Dupuytren’s fibroblasts [25]. This demonstrates that the ECM is not a passive bystander but actively interferes with canonical Wnt/β-catenin signaling, one of the key profibrotic pathways implicated in DD. Similarly, fibronectin has been shown to stimulate myofibroblast differentiation and to synergize with TGF-β1 in promoting α-SMA expression [26]. Together, these results underscore the bidirectional corss-talk between fibroblasts and ECM components, in which ECM remodeling both results from and perpetuates fibroblast activation. Histological analyses of Dupuytren’s tissue corroborate these in vitro findings. Nodules and cords show marked deposition of collagen I, collagen III, fibronectin and tenascin-C, as well as altered distribution of proteoglycans such as decorin and biglycan [26,27]. The altered ratio of collagen I to collagen III, in particular, is thought to contribute to the reduced elasticity of the palmar fascia and to the progressive contracture seen clinically [28]. Another important aspect is the dysregulation of matrix metalloproteinases (MMPs) and their inhibitors (TIMPs). Studies have demonstrated increased TIMP expression and reduced MMP activity in DD tissues, favoring excessive ECM accumulation [29]. This imbalance prevents effective ECM turnover and contributes to the persistence of fibrotic tissue. Altogether, the evidence demonstrates that ECM remodeling in DD is not simply the end result of fibrosis but rather a driver of disease progression, shaping fibroblast behavior through mechanical and biomechanical cues. The continuous cycle of ECM deposition, stiffening and fibroblast activation establishes a feed-forward loop that explains the chronic and recurrent nature of the disease.

## 5. Aberrant Cellular Signaling

Beyond cytogenetic abnormalities and ECM remodeling, aberrant intracellular signaling pathways are a central driver of the fibroproliferative process in DD. Multiple studies included in this review converge on a model in which profibrotic signaling cascades dominate over regulatory or antifibrotic pathways, leading to persistent myofibroblast activation and tissue contracture. TGF-β1 has been consistently identified as a master regulator of fibrosis in DD. In vitro studies demonstrated that stimulation of fibroblasts derived from DD nodules and cords with TGF-β1 induces robust expression of α-smooth muscle actin (αSMA), collagen I and fibronectin, thereby promoting fibroblast-to-myofibroblast differentiation [30]. Pharmacological activation of the cAMP/PKA pathway with forskolin was shown to reverse these profibrotic effects, suppressing αSMA and ECM protein expression [30]. This finding not only reinforces the role of TGF-β1 as a key fibrogenic signal but also highlights intracellular cross-talk between pro- and antifibrotic pathways. Several studies implicated the canonical Wnt/β-catenin pathway in DD pathogenesis. Elevated nuclear β-catenin accumulation was observed in DD fibroblasts compared to controls, correlating with increased contractility and ECM synthesis [8,17]. Interestingly, as discussed in the ECM section, collagen I was found to suppress β-catenin accumulation [25], suggesting that ECM composition modulates Wnt signaling activity. This highlights a complex interplay between the extracellular environment and intracellular signaling cascades. In addition to TGF-β and Wnt, several other pathways have been identified as dysregulated in DD fibroblasts. Activation of MAPK/ERK and PI3K/akt signaling was associated with increased fibroblast proliferation and survival [31]. Notch signaling components were also upregulated, further contributing to fibroblast activation and persistence of the myofibroblast phenotype [32]. Together, these findings illustrate that multiple signaling nodes converge on the common endpoint of sustained fibroblast proliferation, ECM deposition and contractility. A recurrent theme across studies is the integration of multiple signaling inputs. For example, TGF-β1 not only directly induces αSMA expression but also enhances Wnt/β-catenin signaling [32], while integrin-mediated interactions with the ECM further amplify both TGF-β1 and MAPK pathway activation [23]. This extensive cross-talk creates a robust and self-reinforcing signaling network, which explains the persistence and recurrence of fibrosis in DD despite surgical removal of cords.

### Integrated TGF-β/Wnt/ECM Feedback Loop in Dupuytren’s Disease

A coherent picture emerges when considering ECM remodeling, aberrant signaling pathways and fibroblast mechanobiology as interconnected rather than separate domains. TGF-β1 remains a principal inducer of myofibroblast transition, promoting α-SMA incorporation into stress fibers and stimulating collagen I and III synthesis [30,33]. In parallel, the Wnt/β-catenin pathway reinforces cellular proliferation, ECM deposition and cytoskeletal organization, with elevated nuclear β-catenin reported in DD fibroblasts [20]. Notably, these intracellular pathways operate within, and are amplified by, a progressively remodeled ECM. Increased collagen density, altered proteoglycan composition, fibronectin accumulation and reduced MMP activity stiffen the palmar fascia and enhance integrin-mediated mechanotransduction [33]. As fibroblasts perceive elevated matrix stiffness, focal adhesion maturation and RhoA/ROCK activation intensify, further sensitizing cells to TGF-β and Wnt cues [20,30,33,34]. ECM components such as collagen I modulate β-catenin availability, while fibronectin synergizes with TGF-β1 to enhance α-SMA expression [35,36]. This reciprocal reinforcement establishes a self-sustaining feedback circuit: TGF-β and Wnt signaling drive ECM deposition; the remodeled ECM increases mechanical tension, and cytoskeletal forces stabilize the activated state. This integrated loop provides a mechanistic explanation for the chronicity, progression and recurrence of DD and underscores why targeting isolated pathways may be insufficient for durable therapeutic modulation.

## 6. Cytoskeletal Regulation and Contractility

**Figure 1:**
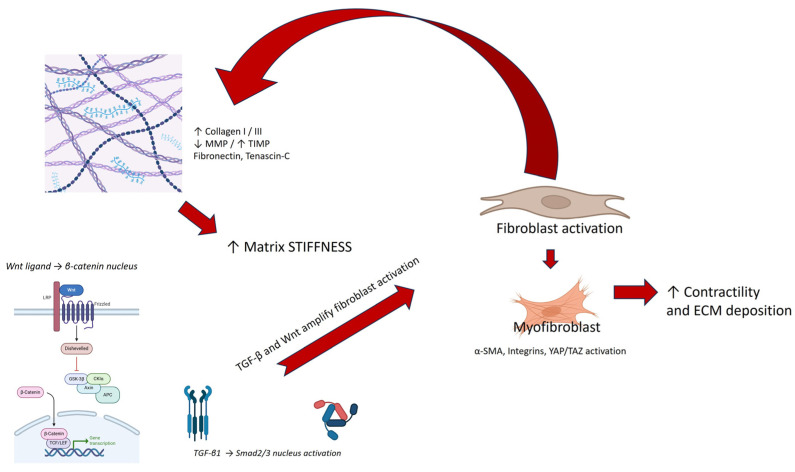
The ECM–cytoskeleton feedback loop in Dupuytren’s disease. Increased collagen I/III deposition and reduced matrix degradation stiffen the fascia, activating integrin-YAP/TAZ and TGF-β/β-catenin signaling in fibroblasts. Enhanced α-SMA-dependent contractility further increases ECM production, generating a self-perpetuating fibrotic loop. Created in BioRender. Pirri, C. (2025) https://BioRender.com/h1cn7w7.

The clinical hallmark of DD is progressive digital flexion contracture, a manifestation of the underlying hypercontractility of palmar fascia fibroblasts. Numerous studies have focused on the cytoskeletal mechanisms that sustain this exaggerated contractile phenotype, highlighting both structural proteins and their molecular regulators. In vitro essays demonstrated that fibroblasts isolated from DD cords exhibit greater contractile capacity compared to fibroblasts derived from adjacent palmar fascia or carpal tunnel controls. This phenomenon has been robustly quantified using collagen gel contraction assays, where DD fibroblasts were shown to reduce gel surface area significantly more than control cells [30,37]. This finding correlates with the clinical progression of nodular thickening into cord formation and eventual contracture. At the molecular level, the upregulation of αSMA is a defining feature of DD fibroblasts. αSMA incorporates into actin stress fibers, increasing intracellular tension and driving contraction of the surrounding ECM [38]. Elevated αSMA expression has been observed in DD nodules and cords compared to normal fascia [30]. Importantly, αSMA not only enhances contractility but also amplifies fibroblast sensitivity to mechanical cues, thereby creating a feedback loop in which tissue stiffness and cellular tension perpetuate one another. One of the most significant insights into cytoskeletal regulation in DD came from the identification of CCT-eta, a submit of the chaperonin-containing RCP-1 complex. Satish et al. [30] demonstrated that DD fibroblasts exhibit overexpression of CCT-eta, which stabilized αSMA filaments and promotes persistent myofibroblast differentiation. Functional studies confirmed that inhibition or silencing of CCT-eta reduces fibroblast contractility, highlighting its role as a molecular switch in sustaining the fibrotic phenotype. This discovery expanded the scope of DD research by linking a protein-folding chaperonin complex to the control of cytoskeletal architecture and contractility. Cytoskeletal regulation in DD cannot be understood in isolation but rather as part of a triangular interplay with signaling pathways and ECM remodeling. TGF-β1 upregulates αSMA expression, ECM stiffness enhances stress fiber formation and Wnt signaling promotes actin organization [8,23]. Pharmacological elevation of cAMP was shown to reduce αSMA expression and weaken contractile activity [30]. This integrated system ensures that fibroblast contractility is both mechanically reinforced and biomechemically sustained (Figure 1).

The persistence of cytoskeletal contractility explains why surgical removal of DD cords is often followed by recurrence: even fibroblasts in “normal”adjacent fascia retain enhanced contractile potential [16]. Moreover, the identification of CCT-eta and αSMA as molecular drivers underscores potential therapeutic opportunities.

## 7. Immune and Inflammatory Crosstalk

While DD has long been viewed as a disorder of fibroblast hyperproliferation and aberrant ECM remodeling, increasing evidence indicates that immune and inflammatory processes play a crucial role in initiating and sustaining fibrosis of the palmar fascia. Several studies included in this review provide consistent evidence of immune cell infiltration, inflammatory mediator release and fibroblast–immune cell cross-talk. Histological analyses of nodular tissue have repeatedly identified infiltrates of mast cells, macrophages and T lymphocytes in proximity to fibroblasts and myofibroblasts [38,39]. Mast cells are of particular interest because they are localized at the interface between fibroblasts and ECM bundles, where they release tryptase, histamine and other mediators that promote fibroblast activation and collagen synthesis. Macrophages have been shown to produce profibrotic cytokines, such as TNF-α and IL-6, which act synergistically with TGF-β1 to induce myofibroblast differentiation [39]. T lymphocytes, especially CD4+ subsets, contribute to the chronic inflammatory milieu, suggesting that adaptive immunity is involved in sustaining disease progression. Immunohistochemical analyses further revealed infiltration by distinct immune cell subsets, including CD45+ leukocytes, CD68+ macrophages and Langherancs cells, localized within Dupuytren’s nodule and cords [10,11]. Their presence indicates that DD lesions are not solely fibroblast-driven but arise within a microenvironment enriched by innate and adaptive immune elements. These immune populations likely act as sentinels and effectors of fibrosis, amplifying the cross-talk between stromal cells and inflammatory mediators. Several studies demonstrated increased levels of pro-inflammatory cytokines, including IL-1β, IL-6, IL-8 and TNF-α, in DD tissue or fibroblast supernatants compared with controls [28,29]. These mediators not only amplify fibroblast activity but also recruit additional immune cells into the lesion, thereby creating a feed-forward inflammatory loop. Moreover, chemokines such as CCL2 and CXCL8 have been implicated in macrophage and neutrophil recruitment, further sustaining inflammation. Functional studies support the view that fibroblasts and immune cells operate in a reciprocal activation cycle. Mast cell degranulation enhances fibroblast contractility, while activated fibroblasts produce cytokines that further recruit and activate immune cells [38]. This cross-talk establishes a chronic profibrotic environment that parallels mechanisms observed in other fibrotic diseases such as pulmonary and hepatic fibrosis.

## 8. Growth Factors and Cytokine Profiles

**Figure 2:**
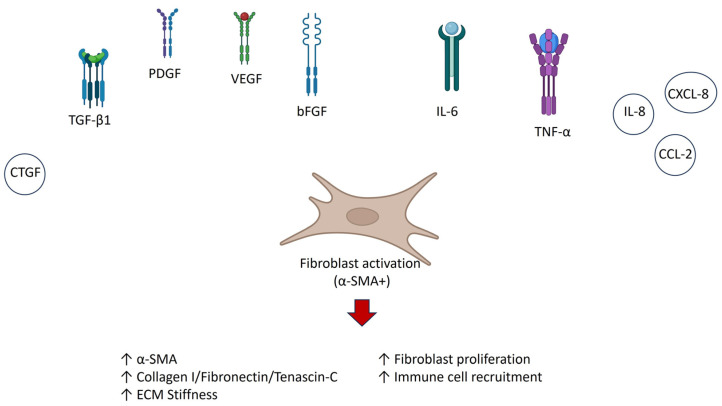
The schematic illustrates the interconnected signaling environment that maintains fibroblast activation within Dupuytren’s nodules. Central myofibroblasts (α-SMA+) respond to elevated growth factors, including TGF-β1, CTGF, PDGF, VEGF and bFGF, which collectively enhance collagen I, fibronectin and tenascin-C synthesis and promote contractility and proliferation. TGF-β1 and CTGF form a synergistic axis amplifying fibrotic remodeling, while PDGF drives fibroblast expansion and VEGF/bFGF facilitate neovascularization and mediator delivery. Concurrently, cytokines and chemokines (IL-6, IL-8, TNF-α, CCL2 and CXCL8) sustain a pro-inflammatory milieu, bridging immune recruitment and fibroblast activation. The resulting cytokine-driven feedback loop reinforces persistent ECM deposition, contractility and inflammation characteristic of Dupuytren’s fibrotic microenvironment. Created in BioRender. Pirri, C. (2025) https://BioRender.com/vwzlkmf.

The pathogenesis of DD is strongly influenced by the local growth factor and cytokine milieu, which shapes fibroblast behavior, ECM deposition and immune activation. A consistent theme across multiple studies is that DD tissue and fibroblasts exist in an environment enriched with profibrotic and pro-inflammatory mediators, creating a niche that drives persistent fibrosis. Among the growth factors, TGF-β1 has emerged as the most potent inducer of myofibroblast differentiation. In vitro stimulation of DD fibroblasts with TGF-β1 results in increased expression of α-SMA, collagen I, fibronectin and tenascin-C [30,36]. These effects were shown to be reversible through pharmacological activation of the cAMP/PKA pathway, underscoring the therapeutic relevance of modulating TGF-β signaling. Closely linked to TGF-β activity is connective tissue growth factor (CTGF), which has been found to be upregulated in DD nodules [37]. CTGF acts downstream of TGF-β and amplifies fibrotic responses, in particular by enhancing ECM production and reinforcing myofibroblast persistence. Together, TGF-β and CTGF form a synergistic axis that drives fibrotic remodeling in DD. Several other growth factors have been detected at elevated levels in DD tissue, including platelet-derived growth factor (PDGF), vascular endothelial growth factor (VEGF) and basic fibroblast growth factor (bFGF) [27,29]. PDGF has been shown to stimulate fibroblast proliferation and migration, thereby contributing to the expansion of the fibrotic lesion. VEGF and bFGF, while classically associated with angiogenesis, may also contribute indirectly to fibrosis by promoting neovascularization within nodules and enhancing delivery of inflammatory cells and mediators to the fibrotic microenvironment. Beyond growth factors, DD fibroblasts and tissue samples have been shown to secrete increased levels of IL-6, IL-8 and TNF-α [30,36]. These cytokines are key mediators of the chronic inflammatory milieu that sustains fibroblast activation. IL-6, in particular, has been implicated as a bridge between inflammation and fibrosis, promoting both immune recruitment and fibroblast differentiation. TNF-α further synergizes with TGF-β in driving myofibroblast persistence, while IL-8 contributes to neutrophil recruitment and localized inflammation. Chemokine profiling has revealed upregulation of molecules such as CCL2 (MCP-1) and CXCL8, which recruit monocytes, macrophages and neutrophils to the lesion [36]. This chemokine-driven influx of immune cells adds an additional layer of amplification to the inflammatory–fibrotic cycle (Figure 2).

## 9. Epigenetic and Molecular Modulators

In addition to cytogenetic abnormalities and dysregulated signaling, an expanding body of research highlights the contribution of epigenetic and molecular regulatory mechanisms to the persistence of fibroblast activation in DD. These mechanisms include altered microRNA expression, histone modification and the activity of regulatory proteins that shape the fibroblast phenotype independently of DNA sequence alterations. Various studies have identified aberrant expression of microRNAs (miRNAs) in DD fibroblasts. miRNAs are small non-coding RNAs that regulate gene expression by targeting mRNA transcripts for degradation or translational repression. In DD, downregulation of the miR-29 family, a well-established regulator of collagen synthesis, has been observed, correlating with elevated collagen type I and III production [36]. Similarly, altered expression of the miR-200 family has been associated with epithelial–mesenchymal transition and myofibroblast persistence [40,41]. These findings suggest that a loss of antifibrotic miRNAs contributes to the fibrotic gene expression program in DD fibroblasts. Epigenetic studies have also revealed alterations in histone acetylation and deacetylation. Overexpression of histone deacetylases (HDACs) has been detected in DD fascial tissue, and pharmacological inhibition of HDACs was shown to attenuate α-SMA expression and fibroblast contractility [24]. These results highlight that fibroblasts in DD may be locked into a fibrotic state through chromatin remodeling, which sustains pathogenic gene expression even in the absence of continuous external stimuli. Moreover, molecular studies have identified oxidative stress as another factor influencing fibroblast behavior. Elevated levels of reactive oxygen species (ROS) were reported in DD fibroblasts, alongside increased expression of antioxidant defense mechanisms [42]. ROS can activate signaling pathways such as MAPK and TGF-β, thereby reinforcing fibroblast activation. In addition to these mechanisms, evidence of mitochondrial dysfunction and oxidative stress has been consistently reported. DD fibroblasts exhibit impaired mitochondrial metabolism and accumulation of ROS, which activate TGF-β/Smad and MAPK pathways, thereby reinforcing fibroblast activation [10,11]. This redox imbalance not only sustains myofibroblast persistence but also suggests a metabolic vulnerability that could be therapeutically exploited. More recently, single-cell transcriptomic analyses have refined our understanding of fibroblast heterogeneity in DD. Distinct subsets of stromal cells expressing PDPN, FAP and TNFRSF12A were identified, displaying high fibrogenic potential and absent in non-diseased fascia [12]. These data demonstrate that DD arises not from a homogenous fibroblast pool but from the expansion of a specialized mesenchymal population uniquely primed for fibrosis. Recognition of this stromal heterogeneity adds a new dimension to our understanding of the disease, highlighting potential cell-specific therapeutic targets. These observations position oxidative stress as both a driver and amplifier of fibrosis. Beyond epigenetics, abnormalities in protein homeostasis have also been implicated. The overexpression of CCT-eta, a molecular chaperonin regulating actin folding, provides a clear example of how alterations in proteostasis contribute to cytoskeletal stabilization and contractility [29]. This finding suggests that disturbances in protein quality control systems may lock fibroblasts into a pathogenic phenotype (Table 2).

## 10. Animal Models and Ex Vivo Systems

Although the majority of mechanistic studies in DD rely on in vitro fibroblast cultures or direct tissue analysis, several investigations have employed animal models and ex vivo systems to better reproduce the complex in vivo environment of the disease. These approaches provide crucial insights into the persistence of fibrosis and the interplay between fibroblasts, ECM and immune components. Ex vivo culture of human DD nodules has proven particularly valuable in maintaining the three-dimensional tissue architecture and cellular heterogeneity of diseased fascia. Karkampuna et al. [38] demonstrated that ex vivo DD tissues preserved fibroblast contractility, ECM deposition and immune infiltration for extended culture periods, thereby offering a more physiologically relevant model than isolated fibroblast clusters. These systems confirmed the presence of mast cells, macrophages and T cells within the tissue microenvironment, reinforcing the concept of fibro-inflammatory cross-talk as a driver of disease progression. Some groups have transplanted human DD tissue into immunocompromised murine hosts to evaluate persistence and remodeling capacity in vivo [23]. Remarkably, transplanted nodules maintained their fibrotic architecture and myofibroblast-rich phenotype, demonstrating that DD fibroblasts possess an intrinsic fibrogenic program independent of the native palmar microenvironment. These xenograft models further revealed that fibroblasts continued to express α-SMA and deposit ECM proteins, indicating that the fibrotic phenotype is self-sustaining and autonomous.

## 11. Discussion

This narrative review included studies spanning cytogenetic analyses, in vitro mechanistic assays, ex vivo models and translational interventions, collectively reframing DD as a fibro-inflammatory disorder with systems-level complexity. The major challenge for interpretation is not the absence of signals but their multiplicity: genetic predisposition, matrix remodeling, signaling imbalance, cytoskeletal “locking”, immune amplification and epigenetic persistence all contribute. A critical analysis must therefore ask why these mechanisms converge, why they sometimes appear contradictory and how they can be leveraged for therapy. A first point of consensus is that fibroblast hypercontractility is the clinical and molecular hallmark of DD. The recurrent demonstration of enhanced gel contraction, upregulated α-SMA and persistent stress fibers [27,30] shows that DD fibroblasts are structurally primed for contraction. Importantly, this is not a transient effect of cytokine exposure but is reinforced by molecular stabilizers such as CCT-eta, which acts as a chaperonin to sustain α-SMA filament stability [27]. This explains why the disease persists even when inflammatory cues diminish: the cytoskeleton itself becomes a memory device. Recent work also highlights the central role of integrins and other ECM receptors in linking extracellular stiffness to cytoskeletal stabilization in DD. Nodule-derived fibroblasts demonstrate increased expression of β1-integrin, enhancing their adhesion to collagen-rich matrices and amplifying mechanosensitive signaling [43]. Engagement of β1-integrin activates focal adhesion kinase (FAK), RhoA/ROCK and downstream MRTF-SRF pathways, each contributing to α-SMA incorporation into stress fibers and the transition toward a stable myofibroblast phenotype [44]. Altered integrin clustering and focal adhesion maturation have been reported in Dupuytren’s fibroblasts, consistent with a heightened sensitivity to matrix rigidity [34]. Moreover, collagen receptors such as DDr1 and CD44 appear upregulated or functionally involved in fibroblast mechanosensing, further strengthening ECM–cytoskeleton coupling and profibrotic behavior [45]. Together, these findings position integrins and related adhesion receptors as key mechanotransducers that convert changes in ECM composition and stiffness into cytoskeletal “locking”, thereby reinforcing the persistence and contractile behavior characteristics of Dupuytren’s disease.

Equally robust is the evidence for matrix–cell reciprocity. The ECM is not a passive bystander but an active regulator of fibroblast behavior. Type I collagen alters β-catenin accumulation and α-SMA expression [22], while pathological fascia shows disrupted collagen ratios, altered proteoglycans and protease imbalance [29,42]. These findings underscore that fibrosis is both the output and the driver of disease: a stiffened ECM sustains myofibroblast activation, creating a feed-forward loop. However, inconsistencies persist, such as collagen suppressing β-catenin in vitro versus reports of robust Wnt activation in other models [7]. These discrepancies likely reflect context-dependence (matrix density, stiffness and dimensionality) rather than true biological conflict, underscoring the need for standardized experimental frameworks. The signaling landscape of DD is dominated by TGF-β1 and Wnt/β-catenin. TGF-β1 has been shown to induce contractile and matrix proteins in DD fibroblasts, effects reversible by forskolin and cAMP/PKA activation [27]. Wnt activation, evident both in genetic association studies and functional assays [7,17], converges on similar endpoints. These pathways interact with MAPK, Notch and PI3K/Akt cascades [46], forming a robust signaling network resistant to perturbation. A critical limitation across studies is the lack of longitudinal data tracking pathway dominance over the natural history of DD, making it difficult to define stage-specific therapeutic windows. The implication is that targeting a single node may not suffice, in particular in late disease. The recognition of an inflammatory component has transformed the conceptual model of DD. Mast cells, macrophages and T cells infiltrate nodules [38], while cytokine signatures (IL-6; TNF-α) reinforce fibroblast activation [2,5]. Functional work identified TNF as a therapeutic target [24], yet later findings demonstrated persistent collagen synthesis despite TNF blockade [47], highlighting a key contradiction between early mechanistic expectations and clinical reality. This divergence is best interpreted as stage-specificity: TNF may be critical during initiation, but once cytoskeletal and matrix circuits are entrenched, upstream blockade is insufficient. Thus, immunomodulation should be part of a temporal strategy, not a standalone solution. Emerging evidence reinforces the view that inflammation plays a decisive and stage-dependent role in the initiation and progression of DD [34]. Early nodules demonstrate abundant infiltration by mast cells, macrophages and T lymphocytes, each contributing distinct profibrotic signals. Mast cells release tryptase, chymase and TGF-β, thereby potentiating myofibroblast differentiation and ECM deposition. Macrophages in DD tissue often exhibit a hybrid M1/M2 phenotype, producing IL-6, TNF-α and profibrotic mediators that synergize with mechanical stress to amplify fibroblast activation. T cells, in particular CD4+ subsets, secrete IL-13 and IL-17, cytokines increasingly recognized as drivers of chronic fibroplasia [41,42]. Inflammatory heterogeneity may shape therapeutic responsiveness, positioning cytokine and immune cell profiling as potential biomarkers for stratification. Of particular importance is the temporal transition from an immune-responsive to a mechanically stabilized state: early lesions remain sensitive to cytokine modulation, whereas established cords become dominated by cytoskeletal and ECM-driven feedback, explaining that anti-TNF agents may reduce inflammation without altering matrix production. These insights highlight inflammation as a dynamic, evolving contributor to fibrosis and a promising axis for stage-specific therapeutic intervention [41,42].

**Figure 3:**
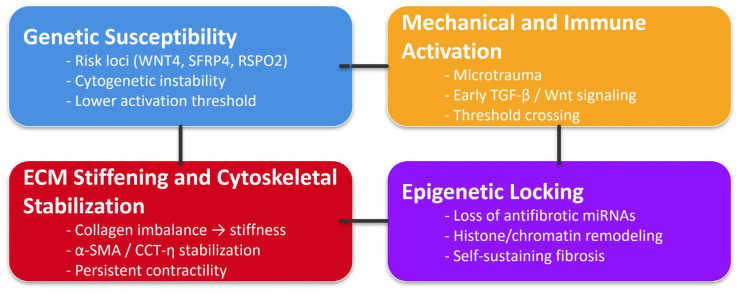
Threshold-and-lock model of Dupuytren’s disease. Genetic susceptibility lowers fibroblast activation thresholds. Mechanical and immune cues trigger TGF-β/Wnt signaling, leading to ECM stiffening and CCT-η-mediated cytoskeletal stabilization. Epigenetic alterations subsequently lock this state, creating a self-sustaining fibrotic loop.

Beyond signaling and immunity, epigenetic and redox mechanisms provide insight into disease persistence. RNA sequencing revealed depletion of collagen-targeting microRNAs [41], while HDAC overexpression and histone modifications contribute to profibrotic gene expression [23]. ROS production and vitamin D deficiency promote TGF-β activation, while antioxidants such as N-acetyl-l-cysteine blunt fibroblast contractility [3,48]. Nevertheless, the literature suffers from limited integration of epigenomic and redox data, with minimal in vivo validation, representing a major knowledge gap in understanding persistence and recurrence. However, these findings shift the focus from transient drivers to the molecular locks that stabilize fibrosis. To further synthesize these converging mechanisms, the present review proposes an integrated conceptual framework describing DD as a “threshold-and-lock disorder”. As illustrated in Figure 3, this model outlines a sequential cascade beginning with genetic susceptibility, incorporating germline risk loci and cytogenetic instability that lower the activation threshold of palmar fibroblasts. Once predisposed cells are exposed to mechanical and immune activation, such as microtrauma, cytokine signaling and early engagement of TGF-β and Wnt pathways, they cross a critical activation boundary that initiates matrix remodeling. This leads to ECM stiffening and CCT-η-mediated cytoskeletal stabilization, whereby altered collagen architecture, increased tissue stiffness and stabilized α-SMA/CCT-η complexes convert transient signals into durable contractility. Finally, epigenetic locking, driven by microRNA dysregulation, histone modifications and redox-dependent TGF-β reinforcement, consolidates a stable fibrotic state that persists independently of the initial triggers. This model extends beyond classical mechanotransduction or epigenetic memory paradigms by explicitly incorporating (i) a threshold dimension, indicating that fibroblasts must accumulate sufficient genetic, mechanical and inflammatory stimuli to cross an irreversible activation point, and (ii) a lock dimension, whereby cytoskeletal stabilization and epigenetic consolidation render the fibrotic phenotype self-sustaining. This integrated view highlights the originality and heuristic value of the “threshold-and-lock” model in explaining persistence, recurrence and therapeutic refractoriness in DD.

Methodological heterogeneity remains the major barrier to synthesis. Small sample size, inconsistent controls (adjacent palmar fascia vs. carpal tunnel tissue) and variable culture systems complicate direct comparisons. For example, palmar fascia in carpal tunnel syndrome may itself harbor fibroproliferative changes, while adjacent fascia may already be “primed”. Likewise, 2D plastic cultures exaggerate some signals while suppressing others compared to biomimetic 3D models [2]. These methodological disparities explain much of the variability in reported signaling outcomes and represent a priority for future standardization.

From a translational standpoint, the synthesis points to several key lessons. First, combinatorial, stage-aware therapies are necessary. Early nodules may benefit from anti-inflammatory and anti-TGF-β strategies [3,23], while late cords may require direct modulation of contractility [30], matrix turnover [48] or epigenetic resets [41]. Second, biomarker-guided stratification could help align therapy with patient-specific pathway dominance: nuclear β-catenin as a marker for Wnt-driven disease, p-Smad2/3 for TGF-β and cytokine panels for inflammatory phenotypes. Third, preclinical platforms must evolve. Ex vivo and xenograft models confirm the intrinsic fibrogenic program of DD fibroblasts [37,49,50], and these should replace simplistic “DD systems” in therapeutic testing.

Furthermore, DD can be understood as a threshold-and-lock disorder. Genetically predisposed fibroblasts [15,51] experience microtrauma or cytokine stress that activates TGF-β and Wnt cascades [30,42]. This triggers ECM deposition and α-SMA/CCT-eta-mediated cytoskeletal stabilization [52,53]. The resulting matrix stiffness and contractility recruit and reinforce immune signaling [5,23,53], while epigenetic and redox programs consolidate the state [41]. Once this loop is established, recurrence and resistance to monotherapy are inevitable unless multiple axes are targeted. Accordingly, negative or inconsistent therapeutic trials should not be interpreted as failures but as boundary conditions that clarify the stages and contexts in which interventions are effective.

The mechanistic architecture of DD offers multiple points of therapeutic and diagnostic intervention. Among the most promising therapeutic targets, TGF-β signaling remains central, and strategies such as ligand blockade, PKA activation or downstream inhibition may attenuate early myofibroblast induction. Epigenetic regulators, in particular histone deacetylases (HDACs), represent another clinical node capable of reversing stable profibrotic gene programs, while CCT-η emerges as a novel target whose inhibition may destabilize α-SMA filaments and disrupt the persistent contractile phenotype characteristic of advanced disease. Parallel advances in biomarker discovery may support patient stratification, with nuclear β-catenin identifying Wnt-driven phenotypes, miR-29 depletion reflecting epigenetic consolidation and quantitative ECM stiffness metrics serving as indicators of disease stage and treatment responsiveness. Future clinical strategies will require multimodal and stage-specific approaches: anti-inflammatory or anti-TGF-β therapies may be most effective in early disease, whereas established cord may require combined modulation of contractility, matrix remodeling and chromatin structure. Integrating molecular biomarkers, imaging-based mechanical assessments and individual genetic profiles could lay the foundation for a precision-medicine framework capable of aligning therapeutic intensity with mechanistic dominance. This translational synthesis delineates a roadmap for converting mechanistic insight into durable therapeutic advances.

### Future Directions

Addressing the unresolved questions in DD will require (1) longitudinal tissue sampling to map temporal transitions in fibroblast phenotypes; (2) the development of physiologically relevant in vivo and organoid models to validate mechanistic hypotheses; (3) harmonization of experimental conditions, including ECM stiffness, culture dimensionality and cell sourcing, to reduce methodological noise; and (4) multimodal, biomarker-guided, stage-specific therapeutic strategies targeting immunity, contractility, matrix remodeling and epigenetic stabilization in combination rather than isolation (Table 3).

## 12. Conclusions

Dupuytren’s disease is a localized but complex fibroproliferative disorder, driven by myofibroblasts activation, immune signaling and metabolic dysregulation. Despite advances in surgical and pharmacological treatments, recurrence remains high, underscoring the need for innovative therapeutic approaches. Recognizing its shared pathways with systemic fibrotic diseases may open opportunities for targeted interventions and biomarker-based strategies. Ultimately, a shift from symptomatic management toward disease-modifying therapies is essential to improve long-term outcomes.

## Figures and Tables

**Table 1:** Cytogenetic and genetic evidence implicating chromosomal instability and heritable risk loci in Dupuytren’s disease.

Category	Main Findings/Alterations	References	Key Message/Implications for DD
Classical cytogenetics (early studies)	Trisomy 8 detected in >50% of DD fibroblast cultures; also observed in some carpal tunnel syndrome controls	[14]	Provided the first evidence that genomic instability may predispose to fibroproliferative activity in the palmar fascia
Subsequent cytogenetic studies	Trisomy 7; deletions of chromosomes 6 and 11; structural rearrangements in nodule-derived fibroblasts	[8,15,16,17]	Although not entirely consistent across studies, these abnormalities support clonal expansion of genetically altered fibroblasts
Heritability/familial clustering	Familial aggregation of DD cases, particularly in Northern European populations	[18]	Supports a strong heritable component in DD susceptibility
Linkage analyses	Implicated regions on chromosomes 16q and 6p, but no single causative gene identified	[19]	Indicates germline susceptibility loci without a clearly defined causal gene
Early GWAS findings	Susceptibility loci identified in Wnt signaling and extracellular matrix (ECM)-related genes	[20]	Demonstrates convergence between genetic risk and profibrotic molecular pathways
Recent GWAS studies	>26 susceptibility loci identified; variants near WNT4, SFRP4 and RSPO2 strongly associated with DD	[8,9,21]	Strengthens the link between inherited predisposition and canonical fibrogenic (Wnt/ECM) signaling pathways
Integrated interpretation	Somatic chromosomal instability combined with inherited genetic predisposition	[8,9,14,15,18,19,20,21]	Chromosomal instability may drive fibroblast proliferation and ECM deposition, while germline predisposition defines at-risk individuals
Shared features with other fibroproliferative disorders	Overlap of chromosomal abnormalities with keloids and Peyronie’s disease	[15]	Suggests a shared pathogenic architecture across fibroproliferative conditions

**Table 2:** Epigenetic and molecular regulatory mechanisms sustaining fibroblast activation in Dupuytren’s disease.

Mechanism/Category	Molecular Players/Markers	Functional Consequence	Pathogenic Relevance in DD	Key References
**microRNA dysregulation**	↓ miR-29 family	Loss of repression of collagen genes (COL1A1, COL3A1)	↑ Collagen I/III synthesis and ECM accumulation	[40]
	↓ miR-200 family	Deregulated epithelial–mesenchymal transition (EMT) and fibroblast persistence	Sustained myofibroblast phenotype	[41]
**Histone modification/chromatin remodeling**	↑ HDAC expression (HDAC1/2/4)	Hypoacetylation of profibrotic gene promoters	Chromatin “locking” of α-SMA and ECM genes → persistent fibrotic state	[24]
**Redox imbalance and oxidative stress**	↑ Reactive oxygen species (ROS)	ROS activation of MAPK and TGF-β/Smad signaling	Reinforcement of fibroblast activation and collagen deposition	[10,11,42]
	↑ Antioxidant enzymes (SOD2, catalase)			
**Mitochondrial dysfunction**	Impaired oxidative phosphorylation, ΔΨm loss	Energy stress and ROS production	Promotes profibrotic signaling and metabolic reprogramming	[10,11]
**Stromal heterogeneity (single-cell studies)**	PDPN^+^, FAP^+^, TNFRSF12A^+^ mesenchymal subsets	Distinct fibrogenic fibroblast populations	Defines cell-specific drivers of fibrosis, absent in normal fascia	[12]
**Proteostasis dysregulation**	↑ CCT-η (chaperonin-containing TCP-1 complex subunit)	Stabilization of α-SMA filaments and actin cytoskeleton	Maintains contractile phenotype and mechanical “memory”	[29]

↑: increase; ↓: decrease.

**Table 3:** Future research directions in Dupuytren’s disease across key mechanistic pathways.

Mechanistic Axis	Unresolved Questions/Knowledge Gaps	Recommended Future Research Directions
**TGF-β signaling**	Lack of longitudinal data on TGF-β dominance across disease stages. Unclear threshold levels that trigger irreversible contractility. Limited in vivo validation of pathway inhibitors.	Prospective tissue studies mapping temporal TGF-β activation (single-cell, phospho-proteomics). Stage-stratified trials of TGF-β modulators (e.g., PKA activators, HDAC inhibitors). Development of in vivo models enabling temporal manipulation of TGF-β cues.
**Wnt/β-Catenin Pathway**	Context-dependent and sometimes contradictory results between ECM conditions and Wnt activation. Genetic risk variants lack functional in vivo interpretation.	Standardized ECM conditions in Wnt assays to clarify biomechanical modulation. CRISPR-based functional studies of risk alleles (WNT4, SFRP4, RSPO2). Use of organoid or xenograft models to test Wnt-targeting strategies.
**ECM Feedback and Mechanotransduction**	Incomplete understanding of stiffness thresholds causing irreversible myofibroblast locking. Heterogeneous ECM characterization across studies.	High-resolution mechanical mapping (AFM, SWE, nanoindentation) of early vs. late lesions. Biomimetic 3D models to define stiffness-dependent signaling switches. Therapeutic testing of matrix-modifying agents (MMP modulators, LOX inhibitors).
**Immune and Inflammatory Circuits**	Stage-specific immune contributions remain poorly defined. Contradictions between TNF-dependent and TNF-independent fibrosis.	Longitudinal immune profiling to identify initiation vs. propagation signals. Stratified trials combining anti-inflammatory agents with cytoskeletal or ECM-directed therapies. Development of immune-competent in vivo DD models.
**Cytoskeletal Stabilization and Contractility**	Mechanisms underlying persistence of stress fibers despite cytokine withdrawal remain incompletely mapped. Limited translational testing of direct contractility modulators.	Molecular dissection of CCT-eta/α-SMA stabilization complexes. Pharmacological screens targeting contractility (ROCK, MRTF, YAP/TAZ). Validation of biomechanical biomarkers predicting contractile phenotypes.
**Epigenetic and Redox Regulation**	Sparse integration of epigenomic, miRNA, and redox datasets. Absence of in vivo epigenetic perturbation studies.	Multi-omics integration (ATAC-seq, miRNA-seq, methylation profiling) to define stable fibrotic programs. Testing HDAC inhibitors, miRNA mimics, or redox modulators in advanced 3D and xenograft models. Identification of epigenetic markers predicting recurrence.
**Translational and Preclinical Models**	Over-reliance on 2D fibroblast cultures; lack of physiological models capturing tissue architecture. No dynamic models of microtrauma-induced initiation.	Development of humanized organoid and bioreactor models. In vivo microtrauma models to test initiation thresholds. Adoption of standardized reporting frameworks for ECM, stiffness, and culture conditions.
**Clinical Stratification and Therapy Design**	Limited biomarkers for distinguishing inflammatory-dominant vs. contractility-dominant phenotypes. Unclear therapeutic windows for early vs. late disease targeting.	Biomarker-guided phenotyping (β-catenin, p-Smad2/3, cytokine panels). Stage-specific therapeutic sequencing (anti-inflammatory → antifibrotic → epigenetic). Multimodal trial designs integrating imaging, molecular profiling, and functional assays.

## Data Availability

No new data were created or analyzed in this study. Data sharing is not applicable to this article.

## Abbreviations

The following abbreviations are used in this manuscript:

DD	Dupuytren’s disease
α-SMA	α-smooth muscle actin
TGF-β1	Transforming growth factor-β1
LD	Linear dichroism
PDGF	Platelet-derived growth factor
PAI-1	Plasminogen Activator Inhibitor-1
IGF-2	Insulin-like Growth Factor 2
WT 1	Wilms Tumor 1
Wnt 4	Wnt Family Member 4
FAK	Focal adhesion kinase
ROCK	Rho-associated coiled-coil containing protein kinase
